# Frontiers on Sustainable Food Packaging

**DOI:** 10.3390/foods12020349

**Published:** 2023-01-11

**Authors:** Rui M. S. Cruz, Theodoros Varzakas

**Affiliations:** 1 Department of Food Engineering, Institute of Engineering, Campus da Penha, Universidade do Algarve, 8005-139 Faro, Portugal; 2 MED—Mediterranean Institute for Agriculture, Environment and Development and CHANGE—Global Change and Sustainability Institute, Faculty of Sciences and Technology, Campus de Gambelas, Universidade do Algarve, 8005-139 Faro, Portugal; 3 Department of Food Science and Technology, University of Peloponnese, 24100 Kalamata, Greece

The implementation of sustainable food packaging solutions within future circular food supply chains is essential to protect customers and ensure food quality, safety, and optimal shelf-life. This will be improved by new innovative packaging materials and will contribute to reducing food waste. In this direction, it is important to employ lifecycle assessment (LCA) to define food supply chain impacts, taking into consideration food waste, global food industry environmental impacts, and shipping distances, with the aim of achieving consumer satisfaction. It is important to share data on (i) the consequences of specific food product–package interactions, (ii) the consideration of the utilization of novel packaging biomaterials, and (iii) overall consumer behavior and satisfaction as a critical focus. The aim of this Special Issue was to bring the most updated information in the new era of sustainability and food packaging.

Dörnyei et al. [1] proposed a literature-based attribute-cue matrix as a tool for analyzing packaging solutions. Using a 2021 snapshot of the wafer market in nine European countries, the study demonstrated the tool’s utility by analyzing the cues found that signal environmentally friendly packaging attributes. Although the literature suggests that environmentally friendly packaging is increasingly used by manufacturers, the analysis of 164 wafer packages showed that communication is very limited except for information related to recyclability and disposal.

The work of Wang et al. [2] presented a supply chain traceability system framework based on blockchain and radio frequency identification (RFID) technology. The system consisted of a decentralized blockchain-enabled data storage platform for data management and an RFID system at the packaging level for data collection and storage. The new traceability system has the potential to simplify the tracking of products and can be scaled for industrial use.

The study of Shin et al. [3] showed the effects of chitosan and duck fat-based emulsion coatings on the quality characteristics and microbial stability of chicken meat during refrigerated storage. The results suggested that chitosan/duck fat-based edible coatings can be used to maintain the quality of raw chicken meat during refrigeration.

Pleva et al. [4] investigated biofilm formation on selected biodegradable polymer films involving selected bacterial strains isolated from dairy products. The antibacterial properties of the films were enhanced with thymol and eugenol. The results showed that these films can be used to prepare novel active food packaging for the dairy industry to prevent biofilm formation and enhance food quality and safety in the future.

Chen et al. [5] developed an edible starch-based film for packaging seasonings in instant noodles. The results showed that the developed starch-based film meets the general requirements of the flavor bag packaging used in instant noodles. Thus, the developed edible film can quickly dissolve into hot water so that the seasoning bag can mix into the soup of instant noodles during preparation. 

López-Gálvez et al. [6] assessed the potential cross-contamination of fresh cauliflowers with *Salmonella enterica* via different contact materials (polypropylene from reusable plastic crates (RPCs), corrugated cardboard, and medium-density fiberboard (MDF) from wooden boxes). The survival of the pathogenic microorganism was studied in cauliflowers and the contact materials during storage. The LCA approach was used to evaluate the environmental impact of produce-handling containers fabricated from the different food-contact materials tested. The results showed a higher risk of cross-contamination via polypropylene compared with cardboard and MDF. Another outcome of the study was the potential of *Salmonella* surviving both in cross-contaminated produce and in contact materials under supply chain conditions. Regarding environmental sustainability, RPCs showed a lower environmental impact than single-use containers (cardboard and wooden boxes).

Cruz et al. [7] presented the environmental impact, trends, and regulatory aspects of bioplastics for food packaging. This review showed that further research is needed to improve the production of bioplastics and their potential applications according to different properties, mechanisms of biodegradation, environmental impacts, markets, and how consumers perceive bioplastics.

In the article of Miller et al. [8] various physical, chemical, and biochemical modifications of potato constituents were identified, and the resulting structural and property changes were presented. The review provided an up-to-date and comprehensive overview of the possibilities and implications of modifying potato components for potential further valorization, particularly in bio-based food packaging. 

The review from Krauter et al. [9] contextualized packaging, sustainability, and related LCA methods. They displayed and discussed how and to what extent food packaging is included in existing LCAs in the cereal and confectionary sector, pointed out the environmental impact of cereal and confectionary packaging in relation to food products with a special focus on GHG emissions, and highlighted improvement strategies to optimize (cereal and confectionary) packaging systems, as well as an LCA of the same. The results revealed that only a few studies sufficiently include (primary, secondary, and tertiary) packaging in LCAs, and when they do, the focus is mainly on their direct (e.g., the material used) rather than indirect environmental impacts (e.g., food losses and waste). 

Bauer et al.’s [10] study aimed at building a comprehensive basis for future sustainable packaging development activities in the area of cereal and confectionary by presenting relevant information on the functions and properties of packaging materials. They detailed product group-specific decay mechanisms and frequently used packaging solutions and highlighted packaging-related shelf-life extension technologies.

In another study, Bauer et al. [11] presented the benefits of multilayer flexible food packaging and showed its negative recyclability trade-offs, especially for food technologists. The review showed that the substitution of non-recyclable flexible barrier packaging is challenging because only a limited number of barriers are available. In the worst case, the restriction on material choice can result in a higher environmental burden through shortened food shelf-life and increased packaging weights.

Junior et al. [12] presented the latest trends in sustainable polymeric food packaging films. This review showed development and advances in bio-based and functional food packaging produced by conventional methodologies and by 3D printing, as well as advances in bio-based alternative feedstock for 3D printing with potential applications in the food packaging area.
